# Cognitive Reappraisal Facilitates Decentering: A Longitudinal Cross-Lagged Analysis Study

**DOI:** 10.3389/fpsyg.2020.00103

**Published:** 2020-01-31

**Authors:** Ryota Kobayashi, Jun Shigematsu, Makoto Miyatani, Takashi Nakao

**Affiliations:** ^1^Department of Psychology, Graduate School of Education, Hiroshima University, Hiroshima, Japan; ^2^Japan Society for the Promotion of Science, Tokyo, Japan

**Keywords:** decentering, cognitive reappraisal, emotion regulation, affect regulation, cross-lagged analysis, longitudinal study

## Abstract

Previous studies have suggested that cognitive reappraisal, which is an effective emotion regulation strategy, enhances decentering. On the other hand, other studies have implied the reverse in regard to this relationship: that decentering supports cognitive reappraisal. However, these possibilities have not yet been examined empirically. In the present study, we investigated the causal relationship between decentering and cognitive reappraisal by conducting two wave cross-lagged analysis. One hundred and thirty-eight Japanese university students responded to a questionnaire comprising measures of decentering and cognitive reappraisal tendency; the questionnaire was administered on two occasions, with an interval of 1 month. Cross-lagged analysis indicated the positive effect of cognitive reappraisal on decentering; however, the reverse possibility, that decentering influences cognitive reappraisal, was not significant. These results suggested that habitual use of cognitive reappraisal fosters decentering.

## Introduction

Several studies of cognitive behavior therapy (CBT) have indicated that “decentering” has beneficial effects on mental health. Decentering is most commonly defined as a metacognitive process in which negative emotions and thoughts are experienced as passing mental events rather than reflections of one’s self or external reality (Teasdale et al., 2002; Bernstein et al., 2015). Previous studies have reported that decentering contributes to reducing depression and preventing its recurrence (Fresco et al., 2007a, b). In addition, decentering has also been shown to contribute to improving anxiety disorders (Arch et al., 2012; Hoge et al., 2015). Specifically, the process by which decentering positively effects mental health is considered to comprise the following: after negative events occur, decentering decreases repetitions of negative thoughts and reactivity to negative thoughts which, in turn, improves mental health (Segal et al., 2002; Teasdale et al., 2002; Bernstein et al., 2015).

Mindfulness-based cognitive therapy (MCBT; Segal et al., 2002; Shapiro et al., 2006) has been found to be effective for fostering decentering. For instance, Bieling et al. (2012) conducted an 8-week mindfulness intervention (comprising mindfulness of breathing and a body scan) and found that MCBT facilitates decentering, which in turn attenuates symptoms of depression. Similar results have been reported in other studies (e.g., Carmody et al., 2009; Hoge et al., 2015). In addition, studies have shown that decentering is enhanced not only by MCBT, but also by other types of CBT; for example, Beck et al.’s (1979) CBT interventions for depression (e.g., Teasdale et al., 2002).

In recent years, the relationship between decentering and cognitive reappraisal, which is one of the most effective emotion regulation strategies used in everyday life, has attracted significant attention from researchers. Emotion regulation refers to attempts to modulate the intensity, duration, and frequency of affect states (Gross, 1998, 2014; Gross et al., 2019). In particular, cognitive reappraisal can be defined as an attempt to reinterpret the meaning of negative emotions, thoughts, or situations (Gross, 2014, 2015); for example, by interpreting events that evoke negative-emotions as educational (Garnefski et al., 2001). Many studies have shown that cognitive reappraisal attenuates negative emotion and promotes mental health (Aldao et al., 2010; Webb et al., 2012; Hu et al., 2014).

Although it has been reported that CBT generally enhances decentering as mentioned above, the possibility that cognitive reappraisal also fosters decentering has recently been suggested. Hayes-Skelton and Graham (2013) proposed this possibility based on consideration of the findings of Fresco et al. (2007b), who revealed that a combination of CBT and cognitive reappraisal training enhances decentering in patients with major depressive disorder. Furthermore, Hayes-Skelton and Graham (2013) also conducted cross-sectional study and reported that habitual use of cognitive reappraisal promotes decentering, which in turn attenuates anxiety.

The following are two mechanisms speculating that cognitive reappraisal facilitates decentering from previous research, though these mechanisms have not yet been examined. First, cognitive reappraisal may involve a process of objectively observing a situation and one’s own state. These processes of cognitive reappraisal may facilitate the distanced perception of mental events that is an important aspect of decentering (Hayes-Skelton and Graham, 2013). Second, implementations of cognitive reappraisal lead to experiences of the process of changing negative emotions and thoughts (Gross, 2014, 2015). Through accumulating these experiences, individuals may understand that emotions and thoughts are only temporary mental events. In this way, cognitive reappraisal may foster decentering (Teasdale et al., 2002).

As mentioned above, there is a possible relationship between cognitive reappraisal and decentering. However, the effect of cognitive reappraisal on decentering has only been reported through cross-sectional surveys. Thus, it remains unclear whether cognitive reappraisal fosters decentering. Therefore, the present study aims to examine the causal relationship between cognitive reappraisal and decentering. To perform this, we conducted a two-wave panel survey and performed cross-lagged model analysis. Further, we created a hypothesis that cognitive reappraisal has a positive cross-lagged effect on decentering based on preliminary evidence (Hayes-Skelton and Graham, 2013).

## Methods

### Participants and Procedure

We administered a specially designed questionnaire to the participants and asked them to answer it at two time points. The second survey (Time 2) was conducted 1 month after the first (Time 1) since previous research has reported that decentering can be changed in at least 1 month (e.g., Josefsson et al., 2014; Fissler et al., 2016). A total of 190 Japanese undergraduate and graduate students answered the questionnaire at Time 1. At Time 2, 141 participants completed the questionnaire. Participants who provided incomplete answers (*n* = 3) or did not answer the survey at Time 2 (*n* = 49) were excluded from the analysis. Therefore, the final analyzed sample comprised 138 participants (62 females). The participants’ average age was 19.08 years (*SD* = 1.45, 18–29). This study was conducted in accordance with the approval of the Research Ethics Committee of Hiroshima University.

### Measures

The Japanese version of the Experiences Questionnaire (EQ; Fresco et al., 2007a; Kurihara et al., 2010) was used to assess decentering. EQ comprises 10 items concerning decentering and five items concerning rumination. Participants provide answers using a five-point Likert scale (1 = “never,” 5 = “all the time”). A sample item from the decentering subscale is “I can observe unpleasant feelings without being drawn into them.” Consistent with previous research (Fresco et al., 2007a), the rumination-related items, which were included as a control against response bias, were excluded from the analysis.

The positive reappraisal subscale of the Cognitive Emotion Regulation Questionnaire (CERQ; Garnefski et al., 2001; Sakakibara, 2015) was used to assess cognitive reappraisal tendency^1^. The positive reappraisal subscale comprises four items (e.g., “I think I can learn something from the situation”). Participants answered using a five-point Likert scale (1 = “never,” 5 = “always”).

### Analyses

We calculated descriptive statistics, Cronbach’s α values, and Pearson’s correlation coefficients using HAD 16.054 (Shimizu, 2016). The cross-lagged model analysis was conducted using maximum likelihood estimation in AMOS version 25.0.0.

## Results

### Descriptive Statics and Correlation Analysis

The means, standard deviations, and Cronbach’s α values for the participants’ responses regarding decentering and cognitive reappraisal are shown in Table 1. Further, the correlations among all variables are also presented in Table 1. Associations between decentering and cognitive reappraisal were significant across surveys (*r*s > 0.35, *p*s < 0.001). The females exhibited lower decentering than did the males, but there was no difference between sexes regarding cognitive reappraisal.

**TABLE 1:** Descriptive statistics and bivariate associations.

	**Mean**	**SD**	**Cronbach’s α**	**1**	**2**	**3**	**4**	**5**
1 T1 Decentering	30.01	5.50	0.78	−				
2 T1 Reappraisal	13.56	3.60	0.83	0.52***	−			
3 T2 Decentering	30.37	5.39	0.80	0.59***	0.44***	−		
4 T2 Reappraisal	13.47	3.26	0.81	0.35***	0.68***	0.57***	−	
5 Age	19.08	1.49	−	0.07	0.10	0.08	0.08	−
6 Sex	−	−	−	−0.19*	–0.03	–0.11	–0.05	0.13

T1, Time 1; T2, Time 2. Sex: Male = 1, Female = 2. ***p < 0.001, *p < 0.05.

### Cross-Lagged Analysis

**FIGURE 1:**
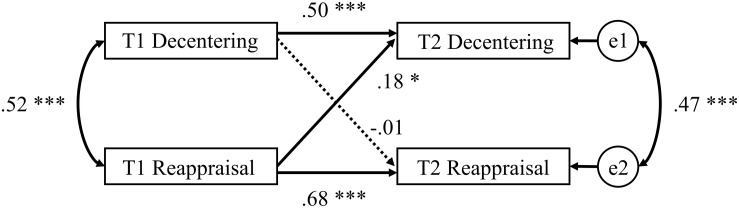
Cross-lagged panel analysis. T1, Time 1; T2, Time 2. Coefficients represent standardized values. The effects of sex and age on decentering and cognitive reappraisal at Time 2 were included, but not shown for ease of presentation. ^∗∗∗^*p* < 0.001, ^∗^*p* < 0.05.

To analyze the causal effect of cognitive reappraisal on decentering, we conducted cross-lagged structural equation modeling, as depicted in Figure 1. In this cross-lagged analysis, we set not only the path from cognitive reappraisal at Time 1 to decentering at Time 2, but also the path from decentering at Time 1 to cognitive reappraisal at Time 2. Moreover, the effects of sex and age on decentering and cognitive reappraisal at Time 2 were controlled in this analysis. The cross-lagged model showed a sufficient fit to the data [χ^2^ (5) = 10.03, *p* = 0.074, CFI = 0.977, GFI = 0.977, RMSEA = 0.086 (90% confidence interval = 0.000–0.163)]. With regard to hypothesis, the cross-lagged effect of cognitive reappraisal at Time 1 on decentering at Time 2 was positive and significant (standardized β = 0.180, standard error (SE) = 0.119, *p* = 0.023). On the other hand, decentering at Time 1 did not influence cognitive reappraisal at Time 2 (standardized β = −0.017, SE = 0.043, *p* = 0.818).

## Discussion

The present study aimed to examine the causal relationship between cognitive reappraisal and decentering. Two wave cross-lagged analysis indicated that people who frequently use cognitive reappraisal are better at decentering, which supports the hypothesis. This positive cross-lagged effect of cognitive reappraisal on decentering can be explained as follows: cognitive reappraisal attenuates negative emotion (e.g., Webb et al., 2012), and leads to distanced perceptions of situations and one’s one states. Therefore, repeated cognitive reappraisal can help an individual understand that emotion and thoughts are only temporal mental events that change over time, and do not necessarily reflect one’s self or external reality (Beck et al., 1979; Teasdale et al., 2002; Beck, 2005). As a result, habitual use of cognitive reappraisal leads to the development of decentering.

On the other hand, the results did not show that decentering influences how often people use cognitive reappraisal. Based on the metacognitive process model of decentering (Bernstein et al., 2015), decentering involves reduced reactivity to thought, emotion, and other mental processes, and thus people with higher decentering may no longer need to perform cognitive reappraisal. Moreover, the measurement of habitual use of cognitive reappraisal in the present study may have influenced the results. Thus, in future study, it will be necessary to experimentally ask participants to perform cognitive reappraisal after inducing decentering and examine whether decentering enhances cognitive reappraisal.

Although the present study focused only on the tendency to perform cognitive reappraisal, cognitive reappraisal can also be considered from another aspect: ability (McRae et al., 2012b; Troy et al., 2013). In this context, tendency means how often a person uses cognitive reappraisal, while ability indicates how well a person can control their negative emotions by employing cognitive reappraisal. As mentioned above, if attenuating negative emotions through cognitive reappraisal influences the effect of cognitive reappraisal on decentering, then both cognitive reappraisal tendency and cognitive reappraisal ability can influence the development of decentering. Thus, the relationship between decentering and cognitive reappraisal ability should be studied further.

### Limitations and Future Directions

The present study has four limitations. First, although the present study conducted a two-wave longitudinal survey, the questionnaire survey method is not sufficient as evidence of causal relationships. Therefore, strong evidence of causality should be examined using experimental methodologies such as experimental intervention. Regarding this point, the causal effect of cognitive reappraisal on decentering can be investigated by performing an intervention that facilitates cognitive reappraisal (e.g., Keng et al., 2016; Shore et al., 2017) and confirming whether decentering is improved as a result.

Second, the possibility that a third variable affected the result of present study cannot be denied. Although the present study indicated the effects of cognitive reappraisal on decentering, perhaps these results may be influenced by other variables such as attention control ability and depressive symptoms. In the future, it should be examined whether the effect of cognitive reappraisal to decentering is observed even in consideration of these variables.

Third, the cognitive reappraisal tendency was measured by the CERQ positive reappraisal subscale (Garnefski et al., 2001; Sakakibara, 2015) in the present study, but another method for measuring cognitive reappraisal should be attempted. The CERQ positive reappraisal subscale seems to be a good scale with regard to reliability and validity. However, it is known that there are several subcategories of cognitive reappraisal (McRae et al., 2012a), and, in particular, the CERQ positive reappraisal subscale measures cognitive reappraisal that re-interprets situations and thoughts “positively.” Thus, in order to examine what kind of cognitive reappraisal affects decentering, it may be necessary to use another cognitive reappraisal scale (e.g., the Emotion Regulation Questionnaire: Gross and John, 2003; the Responses to Stress Questionnaire: Connor-Smith et al., 2000) in the future. In addition, since there are methods that make it possible to measure individual differences of cognitive reappraisal in an experimental task (McRae et al., 2012b; Troy et al., 2013), these methods may be an option in future research.

Fourth, it may be necessary to consider changing the interval length between longitudinal surveys in the future. In this study, based on previous studies (Josefsson et al., 2014; Fissler et al., 2016), the interval between the Time 1 and Time 2 surveys was set to 1 month. However, previous research has also reported that decentering changes over a period longer than 1 month (Teasdale et al., 2002; Fresco et al., 2007b). Hence, it will be necessary to conduct follow-up surveys at intervals of 6 months and/or 1 year, and to examine whether the effect from reappraisal to decentering is observed even across such a time span.

## Data Availability Statement

The datasets generated for this study are available on request to the corresponding author.

## Ethics Statement

The studies involving human participants were reviewed and approved by the Research Ethics Committee of Hiroshima University. The patients/participants provided their written informed consent to participate in this study.

## Author Contributions

RK designed the study, collected and analyzed the data, and prepared the manuscript. JS was involved in the acquisition of the data. JS, MM, and TN reviewed and revised the manuscript. All authors contributed to the manuscript revision, and have read and approved the submitted version.

## Conflict of Interest

The authors declare that the research was conducted in the absence of any commercial or financial relationships that could be construed as a potential conflict of interest.

## Footnotes

1 Although the Emotion Regulation Questionnaire (ERQ; Gross and John, 2003) can be used as a measure of cognitive reappraisal tendency, the Japanese version of ERQ seems to have an item that overlaps with decentering (Sakakibara and Ishii, 2013). As pointed out in previous research (Treynor et al., 2003), there was a possibility that the relationship between decentering and reappraisal might be caused by the influence of such overlapping items. Considering this problem in the Japanese version of ERQ, we used the CERQ positive reappraisal subscale as a measure of cognitive reappraisal.
